# Hepatic Resection *Versus* Transarterial Chemoembolization for Intermediate-Stage Hepatocellular Carcinoma: A Cohort Study

**DOI:** 10.3389/fonc.2021.618937

**Published:** 2021-10-27

**Authors:** Linbin Lu, Peichan Zheng, Zhixian Wu, Xiong Chen

**Affiliations:** ^1^ Department of Oncology, The 900th Hospital of Joint Logistic Support Force, People’s Liberation Army (PLA), Fuzong Clinical College of Fujian Medical University, Fuzhou, China; ^2^ Fujian Center for Safety Evaluation of New Drug, Fujian Medical University, Fuzhou, China; ^3^ Department of Hepatobiliary Disease, The 900th Hospital of Joint Logistic Support Force, People’s Liberation Army (PLA), Fuzong Clinical College of Fujian Medical University, Fuzhou, China

**Keywords:** real-world study (RWS), lactate dehydrogenase (LD), surgical resection, liver cancer (LC), chemoembolization (TACE)

## Abstract

**Background:**

The selection criteria for hepatic resection (HR) in intermediate-stage (IM) hepatocellular carcinoma (HCC) are still controversial. We used real-world data to evaluate the overall survival (OS) in treatment with HR or transarterial chemoembolization (TACE).

**Methods:**

In total, 942 patients with IM-HCC were categorized into the HR group and the TACE group. OS was analyzed using the Kaplan–Meier method, log-rank test, Cox proportional hazards models, and propensity score-matched (PSM) analysis. Curve smoothing was performed through the generalized additive model. The interaction test was performed to evaluate the impact of HR on OS concerning risk factors. Also, we used multiple imputation to deal with missing data.

**Results:**

In total, 23.0% (*n* = 225) of patients received HR. At a median OS of 23.7 months, HR was associated with improved OS in the multivariate analysis [hazard ratio (HzR) = 0.45, 95%CI = 0.35–0.58; after PSM: HzR = 0.56, 95%CI = 0.41–0.77]. Landmark analyses limited to long-term survivors of ≥6 months, ≥1 year, and ≥2 years demonstrated better OS with HR in all subsets (all *p* < 0.05). After PSM analysis, however, HR increased the risk of death by 20% (HzR = 1.20, 95%CI = 0.67–2.15) in the subgroup of patients with lactate dehydrogenase (LDH) ≤192 U/L (*p* for interaction = 0.037). Furthermore, the significant interaction was robust between the LDH and HR with respect to the 1-, 3-, and 5-year observed survival rates (all *p* < 0.05).

**Conclusion:**

HR was superior to TACE for intermediate-stage HCC in patients with LDH levels >192 U/L. Moreover, TACE might be suitable for patients with LDH levels ≤192 U/L.

## Highlights:

- Hepatectomy was superior to transarterial chemoembolization (TACE) for BCLC-B hepatocellular carcinoma (HCC).
- Hepatectomy increased 20% risk of death for LDH <192 U/L after matching.
- A significant interaction was robust between LDH and hepatectomy with respect to the 1-, 3-, and 5-year observed survival rates.

## Introduction

Hepatocellular carcinoma (HCC) is one of the leading causes of cancer-related deaths worldwide and the fifth cause of death in China (1). According to the Barcelona Clinic Liver Cancer (BCLC) staging system, the most widely used scheme, patients with early-stage (stages 0 and A) cancer are suitable for hepatic resection (HR), while intermediate-stage (IM) HCC patients are recommended for transarterial chemoembolization (TACE) (2). Compared with conservative treatment for IM-stage (stage B) HCC, patients treated with TACE have better 2-year overall survival (OS) (3). After selecting the criteria of Bolondi et al. (4), it was shown that patients with stage B1 or B2 cancer have higher 5-year survival rates (21.4% *vs.* 13.9%) (5). Subsequently, the subgroup of IM-HCC patients who benefit from TACE was identified through numerous criteria, including the Assessment for Retreatment with TACE (ART) score (6), the alpha-fetoprotein (AFP), BCLC, Child–Pugh, and response (ABCR) score (7), and the albumin–bilirubin (ALBI) grade (8), among others. Although the highly selected HCC patients have a median survival of 51.5 months (9), the role of TACE is challenged by HR.

A meta-analysis including 18 high-quality studies was recently performed to compare the survival outcomes of 5,986 patients after HR and TACE. The authors found that both stage B and stage C patients showed significantly better OS for HR than for TACE (10). However, a controversial evidence has emerged that HR is superior to TACE only in the subgroup of IM-HCC patients with a lower mortality risk (11–15), such as those in BCLC stages B1/B2 (12, 13). Although the subgroup of IM-HCC patients has been selected using predictive models with a median overall survival (mOS) of 61.3 months, which patients are more suitable for HR is still controversial. Interestingly, Cucchetti et al. (16) performed a regret-based decision curve analysis (Regret-DCA) to choose HR or TACE for IM-HCC patients. In this study, HR should be offered to patients with a 3-year mortality risk <35%, but the optimal strategy (HR *vs.* TACE) is still unclear when the mortality risk is between 35% and 70%. Although numerous subgroups have been identified, more promising biomarkers are urgently needed in order to choose better therapy.

To deal with this issue, we conducted a real-world propensity score-matched cohort study to compare HR and TACE in the treatment of intermediate-stage HCC.

## Methods and Patients

### Patient Selection

The clinical and biological data in our study had been previously published in full (17). In this study, we mainly focus on the derivation cohort from the Sun Yat-sen University Cancer Center (SYSUCC) between January 2007 and May 2012. Details of the inclusion criteria are shown in **Supplementary Figure S1**. A total of 979 patients were included in the derivation cohort. In this cohort, 37 (3.8%) patients were excluded for refusing to receive treatment, and 942 patients were included into the final analysis, with TACE (717/979, 73.2%) or surgical resection (225/979, 23.0%) as the first-line treatment. A total of 805 patients were afforded second-line treatments after the initial treatment at the second follow-up visit (*n* = 597 after TACE; *n* = 208 after HR). According to the decision of the multidisciplinary teams, second-line therapy for these 805 patients included ablative therapies (*n* = 66, 8.2%), surgical resection (*n* = 38, 4.7%), repeated TACE (*n* = 172, 21.4%), other therapies (*n* = 5, 0.6%), or best supportive care (*n* = 524, 65.1%).

The Ethics Committee of SYSUCC approved the study protocol (2017-FXY-129). Because this was a retrospective study, informed consent was waived.

### Diagnosis, Treatment, and Follow-Up

For patients treated with HR, the HCC diagnosis was confirmed by histopathological examination of surgical samples. In contrast, for the patients receiving TACE, the diagnosis was established by the combination of the serum level of alpha-fetoprotein (AFP; over 400 ng/ml) and clinical imaging, which included ultrasonography, computed tomography, or magnetic resonance imaging. If the diagnosis was uncertain based on imaging and the AFP level, a needle biopsy was performed.

Based on the decisions of the multidisciplinary teams, the optimal treatment plan was adopted for each HCC patient. The indications for HR in IM-HCC patients were appropriate residual liver volume determined by computed tomography. For patients without cirrhosis, 30% remnant liver volume after HR was considered adequate. However, for those with chronic hepatitis, cirrhosis, and severe fatty liver, the remnant volume should be more than 50%. Liver resection should not be carried out among intermediate or advanced cirrhosis patients and those with poor liver function (Child–Pugh C). Patients who satisfied the indications for HR were treated by surgical resection, unless the patient requested TACE.

During the initial treatment period, for the first 2 years, patients were followed up every 2 or 3 months to check whether complete remission was achieved. The frequency gradually decreased to every 3–6 months after 2-year remission.

### Variables and Definition

Patients were stratified into a hepatic resection (HR) group and a transarterial chemoembolization (TACE) group. HR was defined as surgical therapy for the lesions in hepatic segments or lobes. Clinically, patients with good liver function and less tumor loading are usually suitable for HR. TACE was defined as chemoembolization of the hepatic artery. The categorical variables consisted of gender, Child–Pugh class (A or B), intrahepatic tumor number (three or less or more than three), and both lobes with lesions (no or yes). Continuous variables, such as age, the diameter of the main tumor, AFP, C-reactive protein (CRP), LDH, hemoglobin (Hgb), white blood cell (WBC) count, and platelet (PLT) level, were also regarded as categorical variables. AFP and PLT were transformed into the Log_10_ scale because of their left skewness. All variables were examined at baseline before any anticancer treatment. The endpoint of interest was OS, which was defined as the time from diagnosis to death by any cause. BCLC stage B and CNLC (China Liver Cancer staging) HCC were defined as follows (18, 19):

BCLC stage B: Two to three lesions, at least one of more than 3 cm in diameter, or more than three lesions of any diameter. Eastern Cooperative Oncology Group (ECOG) PS 0 and Child–Pugh class A or B. Without blood vessel invasion and extrahepatic metastases.

CNLC stage IIa: Two to three lesions, of which at least one is more than 3 cm in diameter. ECOG PS 0–2 and Child–Pugh class A or B. Without blood vessel invasion and extrahepatic metastases.

CNLC stage IIb: More than three lesions of any diameter. ECOG PS 0–2 and Child–Pugh class A or B. Without Blood vessel invasion and extrahepatic metastases.

### Statistical Analyses

To compare differences in the baseline characteristics between the HR and TACE groups, we compared the categorical variables using the chi-square test and the continuous variables using the Mann–Whitney test.

Firstly, survival was calculated using the Kaplan–Meier method, and univariate comparisons were performed using the log-rank test and unadjusted Cox models. Also, multivariable Cox proportional hazards models were adjusted for factors such as the Child–Pugh class, the diameter of the main tumor, location of lesions, intrahepatic tumor number, AFP, LDH, and the PLT level.

Subsequently, to account for potential biases favoring the administration of HR to patients with more favorable baseline prognoses, sequential landmark analyses were performed to evaluate survival with HR or TACE for patients with a minimum of ≥6 months, ≥1 year, and ≥2 years survival from diagnosis. Interaction and stratified analyses were performed for the covariates selected *a priori*, including the Child–Pugh class, diameter of the main tumor, location of lesions, intrahepatic tumor number, CNLC stage, AFP, LDH, and PLT level. To further explore the interaction, curve smoothing was performed between LogLDH and the observed mortality at 1, 3, and 5 years through a generalized additive model.

### Sensitivity Analysis

Finally, we applied three approaches to evaluate the core results in a sensitivity analysis. To minimize potential bias, propensity score (PS)-matched analyses were performed to compare the outcomes of TACE and HR. One-to-one matching (TACE *vs.* HR) without replacement was completed using the nearest-neighbor match on the logit of the PS (derived from age, diameter of the main tumor, location of lesions, intrahepatic tumor number, AFP, Hgb, LDH, WBC, CRP) (all *p* < 0.05 in **Table 1**). The caliper width was 0.02 times the standard deviation of the logit of the PS.

**Table 1:** Baseline characteristics between the transarterial chemoembolization (TACE) and hepatic resection (HR) groups in the derivation cohort.

	Treatment		*p*-value
	TACE (*n* = 717)	HR (*n* = 225)	
Age (years)	53.9 ± 12.3	50.9 ± 12.6	0.001
Gender			0.802
Male	654 (91.2%)	204 (90.7%)	
Female	63 (8.8%)	21 (9.3%)	
HBV infection			0.132*
No	18 (2.8%)	2 (1.0%)	
Yes	622 (97.2%)	203 (99.0%)	
Child–Pugh class			0.302
A	613 (85.5%)	186 (82.7%)	
B	104 (14.5%)	39 (17.3%)	
Diameter of main tumor (cm)	7. 5 ± 3.8	6.4 ± 2.8	<0.001
Location of lesions			<0.001
Unilobar	254 (35.4%)	148 (65.8%)	
Bilobar	463 (64.6%)	77 (34.2%)	
Intrahepatic tumor number			<0.001
≤3	237 (33.1%)	142 (63.1%)	
>3	480 (66.9%)	83 (36.9%)	
AFP (ng/ml)			0.014
<25	180 (26.5%)	75 (35.2%)	
≥25	500 (73.5%)	138 (64.8%)	
CRP (mg/L)			<0.001
<10	318 (45.4%)	72 (32.4%)	
≥10	382 (54.6%)	150 (67.6%)	
Hgb (g/L)			<0.001
<120	148 (20.8%)	71 (31.6%)	
≥120	562 (79.2%)	154 (68.4%)	
LDH (U/L)			<0.001
<245	356 (50.1%)	152 (67.6%)	
≥245	354 (49.9%)	73 (32.4%)	
WBC (10^9^/L)			<0.001
<11	611 (86.9%)	162 (74.0%)	
≥11	92 (13.1%)	57 (26.0%)	
PLT (10^9^/L)			0.249
<150	381 (53.7%)	111 (49.3%)	
≥150	328 (46.3%)	114 (50.7%)	

Numbers that do not add up to 942 are attributable to missing data. Chi-square test was performed for categorical measures and the Kruskal–Wallis test for continuous measures.

HBV, hepatitis B virus; AFP, alpha-fetoprotein; CRP, C-reactive protein; Hgb, hemoglobin; LDH, lactate dehydrogenase; WBC, white blood cell; PLT, platelet.

*Fisher’s exact probability test.

We also used multiple imputation (MI) to maximize statistical power and eliminate bias, which may occur if the confounders with missing data were excluded from the analysis. The MI was based on five replications and the Markov chain Monte Carlo method in the MI procedure in R to account for missing data on Child–Pugh class, diameter of the main tumor, location of lesions, intrahepatic tumor number, PLT, AFP, and LDH. We then created an MI cohort to perform sensitivity analyses using complete-case analysis.

To eliminate the effects of ablative therapies and surgical resection on the second-line treatment, we built a secondary cohort based on the MI cohort without those therapies. All the multivariable Cox analyses mentioned above were repeated in the PS, MI, and secondary cohorts.

Statistical analysis was performed using Empower (X&Y Solutions, Inc., Boston, MA, USA; www.empowerstats.com) and R software (version 3.4.3). A *p*-value <0.05 was considered significant.

## Results

### Descriptive Characteristics

After excluding those who refused to receive treatment (*n* = 33), a total of 942 HCC patients were included in the derivation cohort: 563 patients (59.8%) with CNLC stage IIb (480 patients for TACE and 83 patients for HR) and 379 patients (40.2%) with stage IIa (237 patients for TACE and 142 patients for HR). All patients had good performance status (ECOG PS 0). After first-line treatment with TACE, 46 of 597 patients (6.6%) had invasion of the portal vein or its branch (*n* = 38), hepatic veins (*n* = 6), or of the vena cava/atrium (*n* = 2) and 53 patients (8.9%) had distant metastasis, while 36 patients (6.0%) showed lymph node metastasis at the second follow-up visit.

In the derivation cohort, patients with HR were younger, had shorter diameter of the main tumor, lower hematological indicators (AFP, CRP, Hgb, LDH, and WBC), less frequent intrahepatic tumor number, and with lesions of both lobes (all *p* < 0.05), which are shown in **Table 1**. The majority of the patients (825/942, 87.6%) had hepatitis B virus (HBV) infection, which was treated with nucleos(t)ide analog therapy. The difference in the HBV infection rates was not significant between the HR and TACE groups.

### Survival Analysis for the Entire Cohort

**Figure 1:**
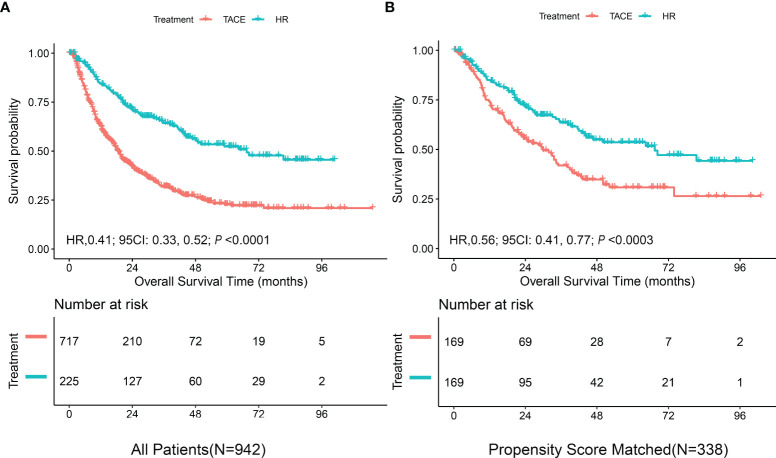
Kaplan–Meier curves of overall survival in the derivation cohort stratified by hepatic resection (HR) and transarterial chemoembolization (TACE). **(A)** All patients. **(B)** Propensity score-matched patients.

As shown in **Figure 1**, the mOS for the entire cohort was 23.7 months (95%CI = 20.4–27.2 months). The mOS rates were 18.5 months (95%CI = 16.9–20.3 months) for the TACE group *versus* 67.4 months (95%CI = 46.7–NA) for the HR group (*p* < 0.0001). After PS matching, the difference in the mOS rates between the TACE (29.9 months, 95%CI = 22.5–38.9) and HR (67.4 months, 95%CI = 44–NA) groups was still significant (*p* < 0.0003).

In the univariable analysis focusing on the entire cohort (**Table 2**), the Child–Pugh class (*vs.* A: HzR = 1.28, 95%CI = 1.01–1.62), diameter of the main tumor (*vs.* <5: HzR = 2.28, 95%CI = 1.86–2.80), location of lesions (*vs.* unilobar: HzR = 1.50, 95%CI = 1.26–1.79), intrahepatic tumor number (*vs.* ≤3: HzR = 1.55, 95%CI = 1.30–1.86), AFP level (*vs.* <25: HzR = 1.63, 95%CI = 1.33–2.00), LDH level (*vs.* <245; HzR = 1.61, 95%CI = 1.36–1.92), and the PLT level (*vs.* <150: HzR = 1.33, 95%CI = 1.12–1.57) were significantly associated with survival (all *p* < 0.05). These variables were included in further analyses.

**Table 2:** Univariate analysis of prognostic factors in the derivation cohort.

	Statistics	Death
Age (years)		
<55	465 (49.36%)	1
≥55	477 (50.64%)	0.95 (0.80–1.13)
Gender		
Male	858 (91.08%)	1
Female	84 (8.92%)	1.15 (0.85–1.56)
Child–Pugh class		
A	799 (84.82%)	1
B	143 (15.18%)	1.28 (1.01–1.62)
Diameter of main tumor (cm)		
<5	300 (31.85%)	1
≥5	642 (68.15%)	2.28 (1.86–2.80)
Lesions of lobe		
Unilobar	402 (42.68%)	1
Bilobar	540 (57.32%)	1.50 (1.26–1.79)
Intrahepatic tumor number		
≤3	379 (40.23%)	1
>3	563 (59.77%)	1.55 (1.30–1.86)
AFP (ng/ml)		
<25	255 (28.56%)	1
≥25	638 (71.44%)	1.63 (1.33–2.00)
CRP (mg/L)		
<10	390 (42.30%)	1
≥10	532 (57.70%)	1.19 (0.99–1.41)
Hgb (g/L)		
<120	219 (23.42%)	1
≥120	716 (76.58%)	0.98 (0.80–1.20)
LDH (U/L)		
<245	508 (54.33%)	1
≥245	427 (45.67%)	1.61 (1.36–1.92)
WBC (10^9^/L)		
<11	773 (83.84%)	1
≥11	149 (16.16%)	1.06 (0.85–1.34)
PLT (10^9^/L)		
<150	492 (52.68%)	1
≥150	442 (47.32%)	1.33 (1.12–1.57)

Numbers that do not add up to 942 are attributable to missing data.

AFP, alpha-fetoprotein; CRP, C-reactive protein; Hgb, hemoglobin; LDH, lactate dehydrogenase; WBC, white blood cell; PLT, platelet.

Subsequently, all seven variables were included in the multivariable analysis shown in **Table 3**. In model I, the adjusted hazard ratio (aHR) was 0.43 (95%CI = 0.34–0.55) for liver resection compared to TACE. To explore the nonlinearity of the confounding factor, the diameter of the main tumor, LogAFP, LDH, and LogPLT were regarded as continuous variables in model II. Compared with TACE, hepatectomy reduced the risk of death by 55% (aHR = 0.45, 95%CI = 0.35–0.58). After PS matching, hepatic resection was still superior to TACE (aHR = 0.56, 95%CI = 0.41–0.77).

**Table 3:** Hepatic resection [*vs.* transarterial chemoembolization (TACE)] and multivariate hazard ratios of overall survival with 95% CIs in Barcelona Clinic Liver Cancer (BCLC) stage B hepatocellular carcinoma (HCC).

	*N* ^a^	Not adjusted	Model I^b^	Model II^a^
Before PS matching	522/876	0.41 (0.33–0.52)	0.43 (0.34–0.55)	0.45 (0.35–0.58)
After MI	553/942	0.41 (0.37–0.46)	0.44 (0.35–0.56)	0.47 (0.37–0.60)
Minus (HR+AT)^c^	382/701	0.39 (0.31–0.51)	0.42 (0.32–0.55)	0.45 (0.34–0.60)
After PS matching	157/338	0.56 (0.41–0.77)	–	–

Numbers that do not add up to 942 are attributable to missing data.

PS, propensity score; MI, multiple imputation; HR, hepatic resection; AT, ablative therapy

a This model was adjusted for Child–Pugh class (A or B), diameter of main tumor (in centimeters), location of lesions (unilobar or bilobar), intrahepatic tumor number (three or less or more than three), LogAFP (in nanograms per milliliter), LDH (in units per liter), and LogPLT (10^9^/L).

b This model was adjusted for Child–Pugh class (A or B), diameter of main tumor (<5 or ≥5 cm), location of lesions (unilobar or bilobar), intrahepatic tumor number (three or less or more than three), and AFP (<25 or ≥25), LDH (<245 or ≥245), and PLT (<150 or ≥150) levels.

c This cohort excluded patients with HR and AT as second-line treatments after MI.

**Figure 2:**
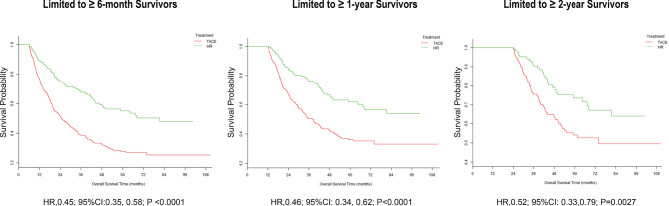
Landmark analyses of overall survival for long-term (≥6 months, ≥1 year, and ≥2 years) survivors.

**Figure 3:**
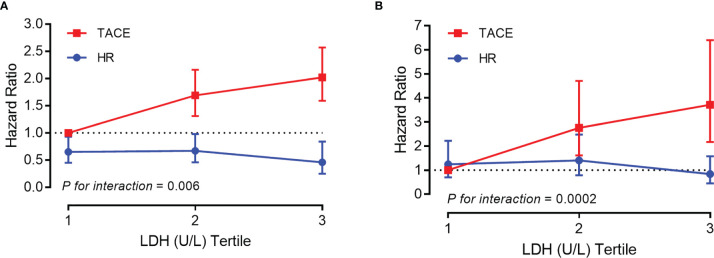
Association between overall survival and platelet count/lactic dehydrogenase stratified by tertile before **(A)** and after **(B)** propensity score (PS) matching [*vs.* transarterial chemoembolization (TACE) in the bottom tertile].

Sequential landmark analysis revealed statistically significant improvement in OS with HR for patients surviving over 6 months (HzR = 0.45, 95%CI = 0.35–0.58), 1 year (HzR = 0.46, 95%CI = 0.34–0.62), and 2 years (HzR = 0.52, 95%CI = 0.33–0.79) (**Figure 2** and **Supplementary Table S2**). In the stratified analyses (**Figure 3** and **Supplementary Tables S3** and **S4**), the magnitude of the association between HR and better survival was more significant for patients with higher LDH (*vs.* the bottom tertile; *p* for interaction = 0.006) and higher PLT (*vs.* the bottom tertile; *p* for interaction = 0.037) levels. After PS matching, however, only for patients with higher LDH levels was there a significant interaction. In the subgroup of patients with LDH <192 U/L (bottom tertile), HR increased the risk of death by 20% (HzR = 1.20, 95%CI = 0.67–2.15). The HzRs were 0.50 (95%CI = 0.30–0.84) and 0.26 (95%CI = 0.14–0.47) in the subgroups of middle tertile (192 < LDH < 255) and top tertile (LDH ≥ 255), respectively. No significant interactions were observed between the effects of TACE and Child–Pugh class, diameter of the main tumor, location of lesions, intrahepatic tumor number, CNLC stage, and AFP.

### Sensitivity Analysis

After MI, HR remained associated with better OS using multivariable Cox regression on the imputed dataset (**Table 3**). The aHRs were 0.44 (95%CI = 0.35–0.56) for model I and 0.47 (95%CI = 0.37–0.60) for model II. Furthermore, the cohort results were still consistent in the MI cohort after excluding the patients with liver resection and ablative therapy as second-line treatments (**Table 3**).

After PS matching of the dataset of derivation cohort, there were no significant differences between the HR and TACE groups (both groups, *n* = 169), as shown in **Supplementary Table S1** and **Figure S2**. The median survival in hepatic resection patients was 67.4 months (95%CI = 44–NA) and that in TACE patients was 29.9 months (95%CI = 22.5–38.9 months). Compared with TACE, liver resection continued to be associated with improved OS (HzR = 0.56, 95%CI = 0.41–0.77, *p* < 0.0003) (**Figure 1B**). The C-statistic of the receiver operating characteristic (ROC)-calculated PS was 0.66 (95%CI = 0.60–0.72).

**Figure 4:**
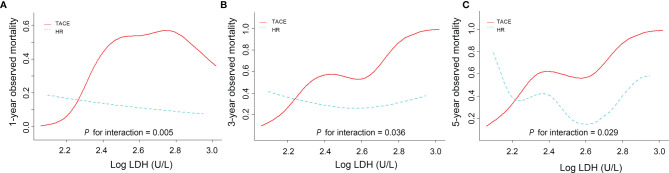
Curve smoothing between LogLDH and observed mortality at 1 **(A)**, 3 **(B)**, and 5 **(C)**  years stratified by hepatic resection (HR) and transarterial chemoembolization (TACE).

Besides, the 1-, 3-, and 5-year observed survival rates were 76.9%, 52.7%, and 46.7% for the TACE group and were 85.8%, 68.6%, and 63.3% for the HR group, respectively. When the LDH level was <192 U/L, however, the mortality rates for HR patients were 2.89 times (95%CI = 0.71–11.81), 1.20 times (95%CI = 0.54–2.65), and 1.22 times (95%CI = 0.57–2.62) *versus* those in the TACE group at 1, 3, and 5 years (**Supplementary Table S4**). The significant interaction was robust between the LDH level and HR concerning the 1-, 3-, and 5-year observed survival rates (all *p* < 0.05) (see **Figure 4** and **Supplementary Table S5**).

## Discussion

In this large-scale, real-world data, we found that the OS for HR was significantly better than that for the TACE counterpart, which was consistent with previous literature (10, 20). Interestingly, Toshifumi et al. (11) also reported that liver resection reduced the risk of death by 44% after PS matching (HzR = 0.56). Notably, this finding remained marked after adjusting for crucial clinical confounders. When the LDH level increased, the magnitude of the association between liver resection and better survival was more significant. After PS matching, however, hepatic resection was associated with worse survival compared with TACE, but not significantly. To the best of our knowledge, this is the first observation of a significant interaction between the effect of HR and the LDH level.

TACE had been recommended as the first-line treatment for unresectable IM-HCC (18). However, whether surgery should be recommended for resectable BCLC-B HCC patients with good liver functional reserve remains a great controversy. In clinical practice from the Asia-Pacific region (21), intrahepatic lesions of more than three tumors, both lobes with tumors, or satellite nodules were not contraindicated for surgical resection of multinodular HCC. Based on the tumor burden, numerous subgroups (11–14) had been identified for the selection of favorable treatments. The previous study showed that a higher LDH level was associated with worse outcomes after hepatectomy or TACE (22). A correlation was also demonstrated between high serum LDH levels and a high tumor volume, a high percentage of necrosis, or an aggressive phenotype for gastric and pancreatic cancer (22, 23). In this study, we found a subgroup in which HR was superior to TACE for IM-HCC: those with LDH levels >192 U/L. Its underlying mechanism is still unclear, and one possible reason might be that surgery reduced the recurrence risk by removing larger lesions with a more aggressive phenotype.

Our study has some strengths. Firstly, we created a propensity score-matched cohort to minimize potential bias. Secondly, our study provided new insights into the selection of appropriate HCC patients for treatment with surgical resection. Hematological indicators, such as the LDH level, should be promising biomarkers.

Our study also has several limitations. Firstly, this is a retrospective cohort with real-world data. Residual bias and unmeasured confounders were unavoidable, even if we had used PS matching to eliminate inherent differences between the two groups. The results after PS matching and MI revealed that the bias from confounders and missing data might have overestimated the advantage of surgical resection. On the contrary, this would make the benefit from TACE treatment more significant in those with LDH levels ≤192 U/L. Secondly, because this is a secondary analysis, the surgical program (radical *vs.* palliative and laparoscopic *vs.* open) was unclear. Differences in the cirrhosis rates, portal hypertension, and the MELD (Model for End-Stage Liver Disease) scores between the two groups were unknown, although the PS matching results were consistent. Thirdly, this study focused on populations from East Asia with hepatitis B between January 2007 and May 2012. Thus, our conclusions might not be applicable to Western populations. With the development of more aggressive surgical treatments, the cutoff value of LDH should be further explored. In the future, the interaction between the effect of HR and the LDH levels should be validated in a randomized control trial and in larger-scale real-world data in various populations.

## Conclusion

Hepatic resection was superior to TACE for intermediate-stage HCC in patients with LDH levels >192 U/L. Moreover, TACE might be suitable for patients with LDH levels ≤192 U/L.

## Data Availability Statement

The original contributions presented in the study are included in the article/**Supplementary Material**. Further inquiries can be directed to the corresponding author.

## Ethics Statement

The studies involving human participants were reviewed and approved by the Ethics Committee of Sun Yat-sen University Cancer Center, which approved the study protocol (2017-FXY-129). Written informed consent for participation was not required for this study, in accordance with the national legislation and the institutional requirements.

## Author Contributions

All authors made a significant contribution to the work reported, whether that is in the conception, study design, execution, acquisition of data, analysis and interpretation, or in all these areas; took part in the drafting, revising, or critically reviewing the article; gave final approval of the version to be published; have agreed on the journal to which the article has been submitted; and agreed to be accountable for all aspects of the work. All authors contributed to the article and approved the submitted version.

## Funding

XC received financial support from the Natural Science Foundation of Fujian Province (nos. 2018J01352, 2016J01576, and 2016J01586) and the Science and Technology Innovation Joint Foundation of Fujian Province (no. 2017Y9125). The funders had no role in the study design, data collection and analysis, decision to publish, or preparation of the manuscript.

## Conflict of Interest

The authors declare that the research was conducted in the absence of any commercial or financial relationships that could be construed as a potential conflict of interest.

## Publisher’s Note

All claims expressed in this article are solely those of the authors and do not necessarily represent those of their affiliated organizations, or those of the publisher, the editors and the reviewers. Any product that may be evaluated in this article, or claim that may be made by its manufacturer, is not guaranteed or endorsed by the publisher.
